# The best internal structure of the Diabetes Quality of Life Measure (DQOL) in Brazilian patients

**DOI:** 10.1186/s12889-024-18090-z

**Published:** 2024-02-23

**Authors:** Denilson Menezes Almeida, Aldair Darlan Santos-de-Araújo, José Mário Costa Brito Júnior, Marcela Cacere, André Pontes-Silva, Cyrene Piazera Costa, Maria Cláudia Gonçalves, José Márcio Soares Leite, Almir Vieira Dibai-Filho, Daniela Bassi-Dibai

**Affiliations:** 1 Postgraduate Program in Management of Health Programs and Services, Universidade Ceuma, Rua Josué Montello, 1, Jardim Renascença. Zip Code, 65075-120 São Luís, Brazil; 2 Department of Physical Therapy, Universidade Federal de São Carlos, São Carlos, Brazil; 3 Department of Physical Therapy, Universidade Ceuma, São Luís, Brazil; 4 Postgraduate Program in Physical Education, Universidade Federal do Maranhão, São Luís, Brazil; 5 Postgraduate Program in Dentistry, Universidade Ceuma, São Luís, Brazil; 6 Postgraduate Program in Environment, Universidade Ceuma, São Luís, Brazil; 7 Department of Medicine, Universidade Ceuma, São Luís, Brazil

**Keywords:** Diabetes Mellitus, Questionnaire structural validation, Quality of life in diabetics

## Abstract

**Background:**

Diabetes Mellitus (DM) is considered a chronic disease with numerous secondary complications that negatively affect the quality of life of patients. However, the specific, known and validated instruments for Brazilian Portuguese are too extensive, which often makes their use infeasible.

**Objective:**

To validate the internal structure of the Brazilian version of the Diabetes Quality of Life (DQOL) measure.

**Methodology:**

Patients with DM type 1 or 2, between the ages of 18 and 76, were evaluated between April 2022 and May 2022. The survey was conducted online using the Google Forms platform. The original DQOL contains 46 multiple-choice questions organized into four domains. For structural validity, confirmatory factor analysis (CFA) was performed using RStudio software (Boston, MA, USA) with the packages lavaan and semPlot.

**Results:**

A total of 354 subjects were evaluated. The 3-domain, 24-item version of the DQOL was the most adequate, with acceptable values for all fit indices (chi-square/GL < 3, TLI and CFI > 0.90, and RMSEA and SRMR < 0.08).

**Conclusion:**

The structure with three domains and 24 items is the most appropriate based on factor analysis. The Brazilian version of the DQOL with a structure of 3 domains and 24 items has adequate measurement properties that support its use in the clinical and scientific context in patients with DM.

## Introduction

Science has made significant advances each year in the identification, screening and targeting of more effective therapies in the prevention of adverse outcomes and the efficient glycemic control of diabetic patients; however, considering the multisystemic influence of the disease, aspects such as activities of daily living, occupation and social relationships are often affected due to organ system complications and impaired quality of life, even leading to non-adherence to treatment [1, 2]. Neglected for many years due to the therapeutic model of treatment of the pathology focused primarily on glycemic control, the quality of life of this population has been the object of interest in several studies and numerous factors have been identified capable of contributing to the worsening of this outcome, such as the presence of the pathology itself, psychosocial aspects, associated comorbidities, type of disease and even demographic and socioeconomic aspects [3, 4].

Several instruments with different orientations and approaches have been developed to assess the quality of life in this population; however, the choice of an adequate and accurate instrument to assess the proposed outcomes can generate difficulties in selecting the best instrument, since to be considered adequate, its measurement properties must have been thoroughly studied and proven to avoid bias [5, 6].

In this context, the Diabetes Quality of Life Measure (DQOL) has become a valid and widely used instrument to assess the quality of life of patients with type 1 and type 2 diabetes mellitus (DM), due to its content validity by experts, good reliability and adequate internal consistency [7]. The Brazilian version of this instrument is originally composed of 44 questions [8], however, although this Brazilian version showed good internal consistency, good discriminant validity (detection of a difference in the perception of quality of life between patients with HbA1c above and below 9%), good correlation between the scores obtained in each domain and convergent validity, its internal structure was not properly evaluated, since the authors did not perform factor analysis.

Thus, a measurement property to be verified in the Brazilian version of the DQOL is the structural validity, in order to determine the degree to which the internal structure of this instrument is adequate for the construct to be measured [8, 9]. Aware of this gap, the discriminatory capacity of the questionnaire, the importance and its wide use in clinical and scientific contexts, in addition to its potential contribution in therapeutic decision-making, this questionnaire was not subjected to a rigorous factor analysis, raising the hypothesis of the possibility that this instrument has adequate internal structural validity in what it proposes to investigate. Parallel to this hypothesis, this study aims to identify the best internal structure of the Brazilian version of the DQOL.

## Methodology

### Study design and ethical aspects

An observational study to assess the psychometric properties of the DQOL, conducted according to the Consensus-based Standards for the Selection of Health Measurement Instruments (COSMIN) [9]. This study was conducted through face-to-face collection in health units in São Luís (Maranhão, Northeastern Brazil) and through the online platform Google Forms (Mountain View, CA, USA). The study procedures were approved by the Research Ethics Committee of the Universidade Ceuma (process number 2.853.570) and conducted according to the Declaration of Helsinki. All subjects were informed of the purpose and procedures of the study, and informed consent was obtained prior to participation.

### Sample size and participants

Recruitment of the volunteers took place through verbal contact, posters and social media. All volunteers included in the study validated their participation by signing or electronically consenting on the free and informed consent form. The sample size was defined as 7 patients for each item on the scale [10]. Considering the DQOL has 46 items, the appropriate minimum number established was 322 patients.

The inclusion criteria were: patients aged 18 years or over, both sexes, clinically diagnosed with type 1 or 2 diabetes mellitus. While the exclusion criteria were: those who had any conditions that prevented them from answering the proposed questionnaire.

### Diabetes quality of life measure (DQOL)

The original Brazilian version of the DQOL contains 44 multiple-choice questions organized into four domains: satisfaction (15 questions), impact (18 questions), social/vocational worry (7 questions) and diabetes-related worry (4 questions). The answers being arranged on a 5-point Likert scale (DCCT *research group*, 1988) [7]. Satisfaction domain: Very satisfied (1)– Quite satisfied (2)– Medium satisfied (3)– Somewhat satisfied (4)– Not at all satisfied (5); Impact domain: Never (1)– Almost never (2)– Sometimes (3)– Almost always (4)– Always (5); Social/vocational worry domain: Never (1)– Almost never (2)– Sometimes (3)– Almost always (4)– Always (5); diabetes-related worry domain: Never (1)– Almost never (2)– Sometimes (3)– Almost always (4)– Always (5). The total score for domain varies from 1 to 5, after adding the answers and dividing by the number of items in the domain. The higher your score, the worse the quality of life.

### Statistical analysis

Data were described as mean and standard deviation (quantitative data) or as absolute numbers and percentages (qualitative data). For structural validity, confirmatory factor analysis (CFA) was performed using the RStudio software (Boston, MA, USA), using the packages lavaan and semPlot. CFA was performed with the implementation of a polychoric matrix and the robust diagonally weighted least squares (RDWLS) extraction method. The model fit was evaluated by the following indices: root mean square error of approximation (RMSEA) with 90% confidence interval (CI), comparative fit index (CFI), Tucker-Lewis Index (TLI), standardized root mean square residual (SRMR), and chi-square/degrees of freedom (DF). Values greater than 0.90 were considered adequate for CFI and TLI, and values less than 0.08 were considered adequate for RMSEA and SRMR. Values below 3.00 were considered adequate in the interpretation of the chi-square/DF [11, 12]. In CFA, factor loadings equal to or greater than 0.40 will be considered adequate for the domain.

The following indices were used to compare the DQOL models, i.e. the original version of the questionnaire [8] and the version proposed in the previous study [13]: Akaike Information Criterion (AIC) and Bayesian Information Criterion (BIC). The model with the lowest AIC and BIC values was considered the best model. For criterion validity analysis, the scores of the long (original) and short versions were correlated, with values ≥ 0.70 considered adequate [14].

## Results

A total of 354 volunteers participated in the study. The majority of the sample consisted of women who were over 50 years of age, overweight, married, non-smokers, and diagnosed with type 2 DM. Additional information about the characteristics of the sample is described in Table 1.

**Table 1:** Characterization of the sample (*n* = 354)

Variables	Mean (standard deviation) or n (%)
Age (years)	51.89 (19.25)
Gender (women)	309 (87.3%)
Body mass (kg)	70.71 (9.86)
Stature (m)	1.65 (0.07)
Body mass index (kg/m²)	25.79 (3.33)
Marital status	
Single	110 (31.1%)
Married	192 (54.2%)
Divorced	37 (10.4%)
Widower	15 (4.3%)
Smoker (yes)	38 (10.7%)
**Associated comorbidities**	
Kidney disease (yes)	28 (7.9%)
Arterial hypertension (yes)	184 (52%)
Heart disease (yes)	38 (10.7%)
Type of diabetes	
Type 1	76 (21.5%)
Type 2	278 (78.5%)
**Short version of DQOL**	
D1 (score, 1–5)	3.28 (0.89)
D2 (score, 1–5)	2.69 (0.93)
D3 (score, 1–5)	2.40 (0.80)
**Long version of DQOL**	
D1 (score, 1–5)	3.35 (0.84)
D2 (score, 1–5)	2.89 (0.83)
D3 (score, 1–5)	2.05 (0.97)
D4 (score, 1–5)	2.82 (0.96)

DQOL: Diabetes Quality of Life Measure; Short version of the DQOL, D1: Satisfaction domain, D2: Impact domain, D3: Worry domain; Long version of DQOL: D1: Satisfaction domain; D2: Impact domain, D3: Social/vocational worry domain, D4: Diabetes-related worry domain

**Fig. 1:**
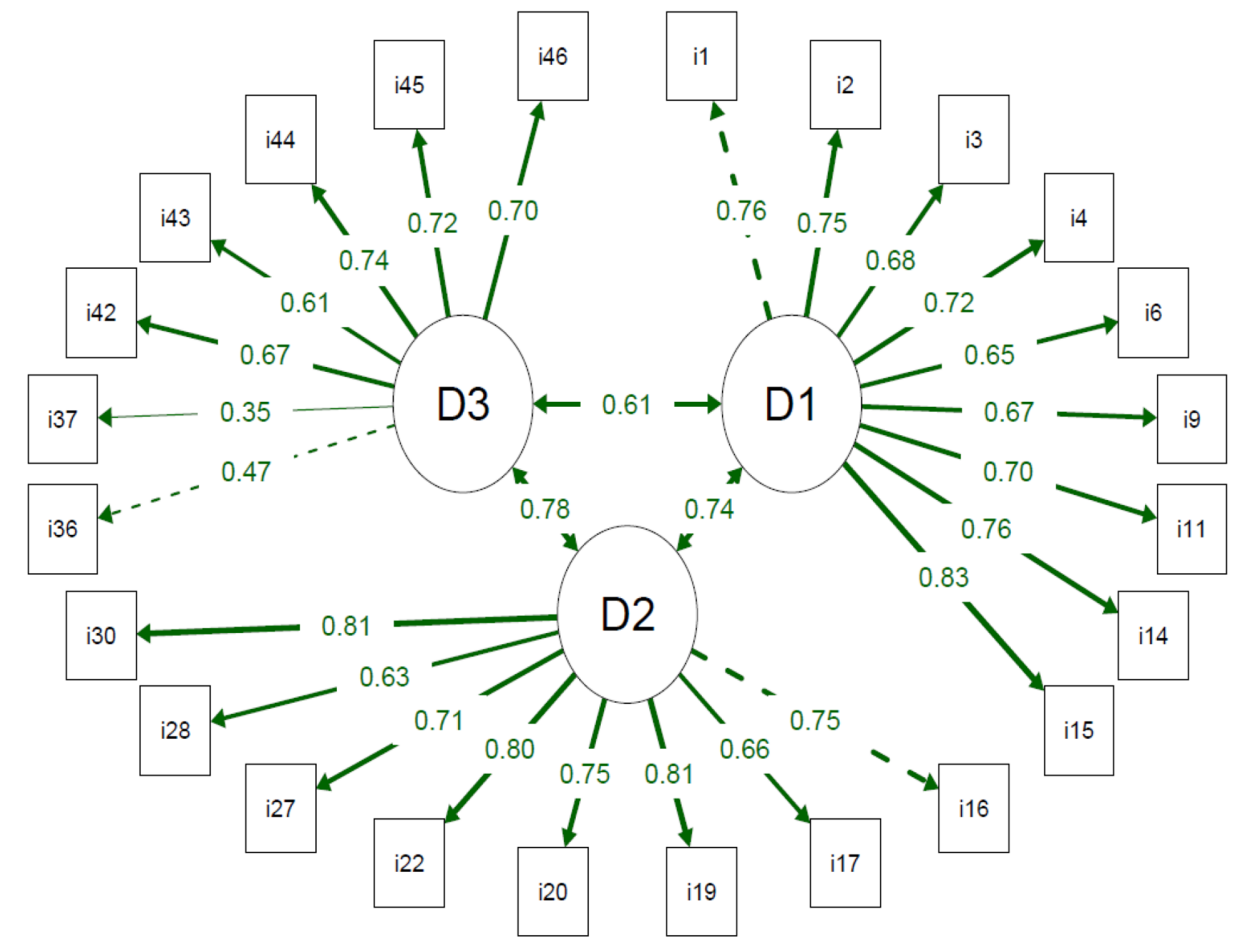
Path diagram of the reduced version of the Diabetes Quality of Life Measure (DQOL). All factor loadings are greater than 0.30. The dotted line indicates the first factor item. The thicker the line, the greater the factorial load. D1: satisfaction domain; D2: impact domain; D3: concern domain

In the structural analysis (Table 2), we found that the long version of the DQOL with 4 domains and 44 items presented inadequate values for CFI and TLI. In contrast, the short version with 3 domains and 24 items showed all adequate fit indices (i.e., chi-square/GL < 3, TLI and CFI > 0.90, and RMSEA and SRMR < 0.08) and factor loadings greater than 0.40 (Fig. 1), in addition to the lowest AIC and BIC values. In addition, the short version has a valid structure and is properly correlated with the long version (rho > 0.76), meeting criterion validity (Table 3).

**Table 2:** Comparison between different structures of Diabetes Quality of Life Measure (DQOL).

Model	Chi-square/DF	CFI	TLI	RMSEA (90% CI)	SRMR	AIC	BIC
Model 1	2.96	0.874	0.867	0.075 (0.071, 0.078)	0.079	46497.167	46860.881
Model 2	2.81	0.941	0.934	0.072 (0.066, 0.078)	0.069	25266.707	25464.041

DF: degrees of freedom; CFI: comparative fit index; TLI: Tucker-Lewis Index; RMSEA: root mean square error of approximation; CI: confidence interval; SRMR: standardized root mean square residual; AIC: Akaike information criterion; BIC: Bayesian information criterion. Model 1: Original Brazilian structure with 4 domains and 44 items; Model 2: reduced structure with 3 domains and 24 items. Chi-square/DF < 3, TLI e CFI > 0.90, e RMSEA e SRMR < 0.08 indicate adequate adjustment of the model

**Table 3:** Correlation between the domains of the original and short version of the Diabetes Quality of Life Measure (DQOL).

Original version DQOL	Short version DQOL		
	D1	D2	D3
D1	rho = 0.966 *	-	-
D2	-	rho = 0.952 *	-
D3	-	-	rho = 0.763 *
D4	-	-	rho = 0.896 *

Short version DQOL, D1: satisfaction domain, D2: impact domain, D3: worry domain; DQOL long version: D1: satisfaction domain; D2: Impact domain, D3: social/vocational worry domain, D4: diabetes-related worry domain. * Significant correlation (*p* < 0.05) using Spearman correlation coefficient (rho). Adequate criterion validity (rho > 0.70)

The short version of the DQOL into Brazilian Portuguese is available at https://questionariosbrasil.blogspot.com/.

## Discussion

The present study evaluated the internal structure of the Brazilian version of the DQOL and showed that a structure of 3 domains and 24 items is adequate for assessing the quality of life. In this short version, items from domain 3 (social/vocational worry) and domain 4 (diabetes-related worry) were grouped into a single domain (worry).

The short version with 3 domains and 24 items presented in the present study was based on the internal structure of the DQOL found in the robust previous Chinese study composed of 2886 patients with diabetes, in which classical test theory (CTT) and item response theory (IRT) were used as reduction methods, each combined with exploratory factor analysis (EFA). Furthermore, the authors used CFA and Spearman correlation coefficient to validate the short version [13].

The DQOL has already been translated and validated in different countries such as Malaysia [15], China [16], Türkiye [17], Spain [18], Arabia [19], Iran [20], Pakistan [21], and Brazil [8]. However, most validations have focused on cross-cultural adaptation, internal consistency, reliability, and construct validity, but not on the structural validity of the instrument. Thus, unlike the present study, the Iranian version tested the original version of the DQOL and found adequate fit indices for the structure with 4 domains and 46 items [20]. When comparing the fit indices, the values from the Iranian study are very similar to those we found in the short version of the DQOL (RMSEA = 0.07, CFI = 0.94 and TLI = 0.93).

Regarding the use of short versions of questionnaires and scales, a previous study points out the main positive points: shorter time to complete the instrument; less possibility of unanswered items; and less possibility of filling errors or random filling of items [22]. Thus, decreasing the size of the DQOL from 44 to 24 items optimizes the response time and reduces the burden on the respondent, leading to greater adherence to the questionnaire, in addition to generating greater clarity and ease in its application.

Strengths of the present study are: (1) the adequate sample size and the analysis conducted according to COSMIN consensus; (2) the implementation of the polychoric correlation matrix when using polytomous data and extraction method of the CFA according to the categorical ordinal nature of DQOL responses. Some limitations must be considered: (1) we conducted the analysis on a sample of Brazilian patients and it is fundamental that future studies with samples from other countries test the DQOL in its version with 3 domains and 24 items; (2) in addition, other measurement properties must also be considered, such as reliability, construct validity and responsiveness.

## Conclusion

The Brazilian version of the DQOL, with a structure of 3 domains and 24 items, has adequate measurement properties that support its use in clinical and scientific contexts in patients with DM type 1 and 2.

### Clinical implications

The length of a questionnaire can contribute to the increase of its non-use and non-response due to the time spent and, often, the loss of interest in the proposed questions, affecting the quality and consistency of the answers. Reducing the size of the DQOL-Brazil from 44 to 24 items optimizes the response time and reduces the burden on the respondent, leading to greater adherence to the questionnaire, in addition to generating greater clarity and ease in its use.

### Strenghts and limitations

Some strengths that strengthen the originality of this investigation deserve to be highlighted, such as: (1) the sample has an adequate size and the analyzes used were conducted in accordance with COSMIN; (2) when using polytomous data, we use a polychoric correlation matrix (ideal for the nature of this analysis). Honestly, we need to highlight some limitations: (1) we conducted the analysis on a sample of Brazilian patients. Therefore, it is essential that future studies with samples from other countries test the DQOL in its version with 3 domains and 24 items; (2) analysis of reliability, construct validity and responsiveness should be considered in future studies.

We used mathematical reasoning (factor analysis) to identify the best internal structure of the DQOL in Brazilian patients, but items 18 and 24 (both from the “worry” domain) do not seem to make sense for a population sample in which more than 50% of patients are married. Therefore, we suggest that further studies should examine whether these findings are replicated in unmarried patients. In addition, we advise clinicians/raters to collect patients’ marital status and analyze whether responses to these items have a significant impact on the DQOL score.

## Data Availability

The set of data generated and/or analyzed during the present study are available through the corresponding author upon reasonable request.
